# Calcitonin Gene–Related Peptide and Thermal Injury: Review of Literature

**Published:** 2009-07-28

**Authors:** Giulio Gherardini, Giuseppe Curinga, Giuseppe Colella, Nicola Freda, Raffaele Rauso

**Affiliations:** ^a^ Plastic Surgeon, Rome, Italy; ^b^ Department of Plastic Surgery-Burn Unit, Civico and Benfratelli Hospital, Palermo, Italy; ^c^ Department of Head and Neck Surgery, Second University of Naples, Italy. Dr Giuseppe Curinga is an ISBI (International Society of Burn Injury) Traveling Fellow currently at Johns Hopkins Hospital in Baltimore, MD.

## Abstract

The aim of this review article is to report about the anti-inflammatory properties of calcitonin gene–related peptide (CGRP) in burns. CGRP is a 37-amino acid neuropeptide, primarily released from sensory nerves and is well known as the most potent and long-lasting microvascular vasodilator in vitro and hypotensive agents in vivo.

A wide range of proinflammatory stimuli can induce the release of neuropeptides from cutaneous sensory nerves, including heat, physical trauma, UV radiation, and irritant chemicals. These proinflammatory stimuli are known to induce the release of CGRP from cutaneous sensory nerves. The anti-inflammatory effects of CGRP in a range of species and in human clinical conditions are detailed, and potential therapeutic applications based on the use of antagonists and gene targeting of agonists are discussed.

Thermal injury of the skin results in local tissue destruction and a systemic response. The increased temperature kills cells in the immediate area and denatures the surrounding extracellular matrix proteins. Earlier experimental investigations have shown that the inflammatory reaction is divided into early and delayed phases. The first phase is believed to be due to the direct effect of heat on burned tissue causing an increased capillary filtration followed by edema formation.^1^ The second, or delayed phase, depends on a cascade of mediators released by the local tissue and central nervous system, leading to physiological changes including increased capillary hydrostatic pressure, leakage of intravascular fluid and proteins into the interstitium, decreased cardiac output, and suppression of the immune system. This delayed reaction is induced by the contribution of sensory neuropeptides such as calcitonin gene–related peptide (CGRP).^2^

CGRP is a 37-amino acid neuropeptide encoded by an alternative processing of the calcitonin gene in thyroid C cells.^3^,^4^ It is found in sensory peptidergic nerves that are present in most tissues and organ systems, including blood vessels, heart, kidney, gastrointestinal system, and lymphoid organs. CGRP is well know as a potent and long-lasting microvascular vasodilator in vitro and a hypotensive agent in vivo.^5^–^11^

CGRP can potentiate inflammatory edema in skin induced by mediators that increase microvascular permeability.^5^ This is thought to be a consequence of its action as a microvascular vasodilator.

CGRP has a proliferative effect on human endothelial cells; therefore, it is important for the formation of new vessels, for example, in ischemia, inflammation, and the healing of wounds; it is also regarded as an important modulator of the inflammatory response after the activation of sensory nerves (Fig 1).^2^,^12^ The aim of this review article is to report the anti-inflammatory properties of CGRP in burns.

## BURN PATHOPHYSIOLOGY

Several investigations have focused on the circulatory and microcirculatory alterations associated with burn shock and edema formation in both burned and nonburned tissues.

Under normal physiological conditions, blood pressure in the capillaries causes the filtration of fluid into the interstitial space. The thermal injury causes extravasation of plasma into the burn wound and the surrounding tissues. Edema develops when the filtration rate exceeds the flow in the lymphatic vessels draining the same tissue. It follows a biphasic pattern. An immediate increase in water is seen in the first hour after injury. A second phase, which is more gradual, occurs 12 to 24 hours after burn injury.^13^ Thermal injury causes direct and indirect mediator-modulated changes of the permeability of blood tissue barrier of the capillaries and venules. This pathophysiological response involves several classes of chemical mediators^14^ interacting in a complex manner to cause the pain and secondary tissue damage (Table 1).

## SENSORY NERVOUS SYSTEM AND INFLAMMATION

There is evidence that the activation of the peripheral nervous system generates the major features of inflammation. The so-called “neurogenic inflammation” is mainly due to the activation of C-fibers^45^ and Aδ-fibers,^46^,^47^ leading to erythema, wheal, and flare.^48^

It is also accepted that these symptoms are due to antidromic release of sensory neuropeptides, in which tachykinins, such as substance P (SP), neurokinin (NK) A, and NKB together with CGRP, play a major role.^48^–^54^

The neurogenic inflammatory response is complex and cannot simply be regarded as a series of neuronal events occurring in isolation. Indeed, it is known that the initiation and sustenance of neurogenic inflammation depend on a variety of factors, such as endothelium, kininogens, and neuropeptides, present in the local environment. The vasodilation response characteristic of neurogenic inflammation requires the presence of endothelium and is linked to the production of nitric oxide (NO).^55^ It has been suggested that NO may act prejunctionally or within peripheral neurons to mediate the release of neuropeptides during neurogenic inflammation within the skin microvasculature.^56^,^57^ In the course of inflammation, ubiquitous kininogens, including bradykinin, are important mediators of inflammation. In addition to direct activation and sensitization of nociceptors, there is evidence that kinins are proinflammatory, leading to vasodilation, plasma extravasation, and the release of other inflammatory mediators, notably the neuropeptides SP and CGRP.^58^ The finding that the pungent extract of the Hungarian pepper, capsaicin (8-methyl-*N*-vanillyl-6-noneamide), can be used as a neurotoxin for the nonmyelinated sensory afferents^59^ is of crucial importance to research on the role of the nervous system in the inflammatory process. Capsaicin is selective for the stimulation and blockage of a subset of mammalian afferent neurons of dorsal root ganglia with C- and Aδ-fibers. In response to stimulation, peptide mediators are released from the central and peripheral nerve endings of these neurons, and both SP and CGRP are involved in the capsaicin-induced reaction. SP induces a short-lasting endothelium-dependent vasodilation through the activation of the NKI receptor,^60^ which is partly dependent on NO release.^61^ SP also causes plasma protein extravasation^62^,^63^ and a concomitant histamine release from mast cells.^64^–^67^

The most prominent features of the neurogenic inflammation are vasodilation. CGRP, most often co-released with SP, is the most potent endogenous vasodilator found in animals and humans^5^,^68^ and the most abundant neuropeptide in the peripheral nervous system.

## CALCITONIN GENE–RELATED PEPTIDE

**Figure 2:**
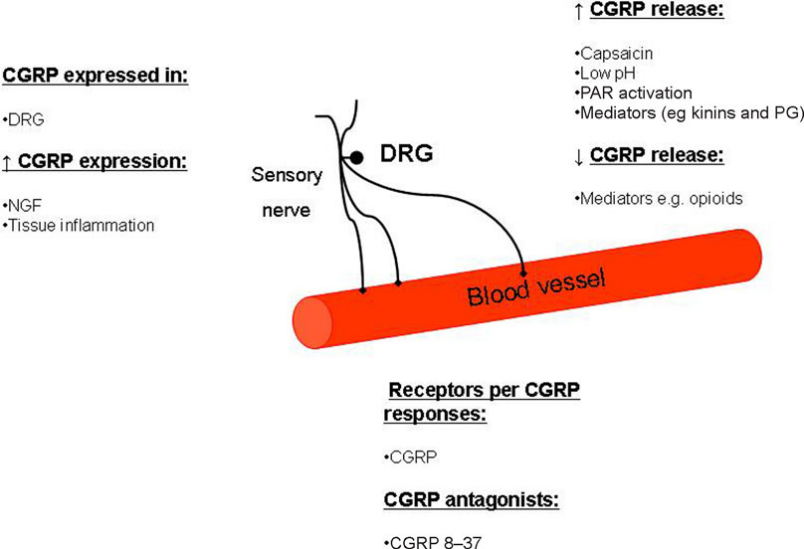
The *CGRP* gene is expressed in the dorsal root ganglion (DRG) and is upregulated by factors that include nerve growth factor (NGF) and tissue inflammation. CGRP is released from nerves in response to several stimuli, such as capsaicin and low pH, proteinase-activated receptor (PAR activation), and mediators (eg, kinins and prostaglandins [PG]). Opioids can inhibit the release of CGRP. The response to CGRP is inhibited by CGRP receptor antagonists

CGRP was identified in 1982 when Rosenfeld et al^4^ showed that alternative RNA processing of the calcitonin gene generated mRNAs encoding a peptide they named CGRP. It is highly expressed in certain nerves and is now known to belong to a family that includes the more recently discovered peptides adrenomedullin and amylin. This group belongs to a larger family of peptides that includes calcitonin. Calcitonin is a potent inhibitor of bone resorption, acting via receptor-mediated inhibition of osteoclast function.^69^ The overall effect of CGRP on bone resorption is unclear, although it can inhibit osteoclast activity,^70^ but it is best known for its potent cardiovascular effects.^71^ CGRP is distributed throughout the central and peripheral nervous systems and exhibits a range of biological effects on tissues including those associated with gastrointestinal, respiratory, endocrine, and central nervous systems (Fig 2).^63^,^72^–^78^

### Vascular system

CGRP is a potent arterial and venous vasodilator and is frequently co-localized with SP. Indeed, SP regulates the vasodilator activity of CGRP,^79^ suggesting that there is an important functional significance to this co-localization. There are several mechanisms by which CGRP produces vascular relaxation, as discussed in earlier reviews^80^–^82^ It is accepted that vasodilation is mediated via the CGRP1 receptor and blocked in a competitive manner by CGRP8–37.

Depending on species and blood vessel type, the vasodilating properties of CGRP can be endothelium dependent or independent, both cases involving an intracellular increase in cAMP.^49^,^83^ In this signaling system, cAMP acts as a second messenger, subsequently activating cAMP-dependent kinase and ultimately regulating ion channels, enzyme activity, and/or structural proteins.^5^,^71^,^84^–^94^

### Inflammation

A wide range of proinflammatory stimuli can induce the release of neuropeptides from cutaneous sensory nerves, including heat, physical trauma, ultraviolet radiation, and irritant chemicals. The release of these neuropeptides leads to neurogenic inflammation with erythema and edema. CGRP is considered to be an important modulator of the inflammatory response after the activation of sensory nerves.^95^ The action of CGRP on edema formation has been extensively studied, and interestingly, CGRP-like immunoreactivity (CGRP-LI) around blood vessels increases in chronic inflammation.^96^ In a series of studies, a potentiating action of CGRP in edema formation was demonstrated, but only when CGRP and SP were administered concomitantly.^5^,^79^,^97^–^99^ Probably, CGRP's potentiation of edema formation is due to its induced vasodilation and not due to its direct effect. The role of CGRP in mast cell degranulation and histamine release has also been studied. The close anatomical relationship between mast cells and sensory nerves in many organs suggests that there is a physiological interaction between these two cell types.^100^ However, the capability of CGRP for the activation of mast cells is less pronounced than that of SP.^48^,^100^ In fact, CGRP has been reported to release little or no histamine.^101^

### Wound healing

It has been postulated that sensory neuropeptides in general act as local growth factors.^102^ There is also increasing evidence that neuropeptides participate in many of the inflammatory processes that are crucial for normal wound healing.^38^ Plasma levels of CGRP are increased in soft tissue injuries^103^ and in patients with chronic cardiac failure and sepsis,^104^ indicating that CGRP may be another important peptide in chronic illness. Animal experiments have shown that rats depleted with sensory neuropeptides show reduced inflammatory responses, as well as poor wound healing and diminished skin integrity.^105^

CGRP seems to be of importance in the formation of new vessels through the induction of endothelial cell proliferation during pathophysiological events such as ischemia, inflammation, and wound healing.^106^ In the survival of ischemic denervated tissue, the importance of reinnervation of mainly CGRP-containing fibers has been stressed.^107^ These data suggest that the healing process is also related to the anti-inflammatory effects of CGRP, and upregulation of CGRP binding sites are reported in selective brain areas (involved in the integration of sensory information) following stress.^108^

### Immune system

Neuropeptides are capable of interacting with almost all components of the immune system. CGRP is a potent anti-inflammatory mediator; it is thought to inhibit type 1 cytokines (eg, interleukin [IL]-12 and interferon γ) and to enhance the production of IL-10, one of the most immunosuppressive cytokines.^109^,^110^ Gomes et al^12^ observed anti-inflammatory effects of CGRP in models of acute peritonitis, reducing the recruitment of neutrophils induced by treatment with lipopolysaccharides. The anti-inflammatory effect of CGRP is comparable with the proinflammatory effects of SP, bradykinin, and endothelin and suggests that different vasoactive peptides could participate in opposite ways on macrophage activation during local and systemic acute inflammation and possibly bacterial sepsis.^12^

## CGRP AND BURNS

The reaction after a local burn injury is dependent on the temperature and duration of the burn.^111^ Several experimental investigations have shown that the delayed phase in burn inflammation is mediated by humoral and neurochemical factors, and pharmacological intervention could therefore be of clinical importance.^112^ The contribution of sensory neuropeptides has been shown in the delayed phase. Several vasoactive neuropeptides have been proposed as mediators of the delayed phase reaction.^113^

Although not necessarily connected, the similarities between neurogenic inflammation and the reactions after a burn injury are intriguing. The role of neurons in the response after a burn injury was first suggested by Sevitt,^111^ who observed that denervated skin showed a higher (2–3°C) threshold temperature at which plasma extravasation developed. By applying a hot iron to the skin, he showed that a specific temperature initially induced an erythema and if the application was prolonged, plasma extravasation could also be observed. With decreasing temperatures, proportionally longer heat exposure was required for the development of edema. In this context, it is interesting to note that the threshold temperature for edema formation has been estimated to 45°C^111^,^114^ and that this is similar to threshold temperature for the activation of nociceptive fibers in the skin.^115^ Furthermore, in more severe burns, edema can also be observed in subdermal structures and this has been correlated to subdermal temperatures of 41°C to 45°C during the time of exposure.^111^ Early and delayed edema formation has been demonstrated in burns of different severity.^111^,^116^ In more severe burns, the early part is rapid in onset and the delayed part is often indistinguishable from the early or abolished part due to stasis produced by the early massive edema formation. In milder burns, the early part is less pronounced and sometimes followed by a distinct delayed increase in edema formation occurring 4 to 8 hours postburn.^111^,^116^ This latter effect can be observed after a well-defined exposure of the skin to hot water (60°C, 10 seconds).^116^

The first evidence for the importance of nociceptive C-fibers was obtained in 1983. It has been shown that capsaicin pretreatment reduced edema formation after a mild (48°C, 5 minutes) scalding injury,^117^ and that this edema was also reduced by an SP antagonist but not by antihistamines.^118^,^119^

Burn injury leads to the release of SP and CGRP from nociceptive sensory endings.^120^,^121^ CGRP and SP contribute to the spread of edema by acting directly on venules to produce vasodilation. CGRP affects the regulation of local blood flow, smooth muscle tone, and glandular secretion. Siney and Brain^122^ confirmed these findings in rat dorsal skin by the use of selective SP and CGRP antagonists. SP and CGRP also seem to play a role in the initial plasma extravasation observed after thermal injury in rat dorsal skin.^122^

SP has a major role in the initial plasma extravasation after injury. Moreover, CGRP is involved in mediating plasma extravasation for up to 15 minutes after the onset of thermal injury.^122^ Löfgren et al^123^ demonstrated increased concentrations of CGRP-LI in a perfused rat paw following thermal injury, and thermal injury resulted in a unilateral increase in blood flow paralleled by an increased content of CGRP-LI and NKA-like immunoreactivity in paw perfusate.^124^ The thermally induced inflammation of the rat paw caused locally increased perfusion, which was characterized by 2 phases. Notably, the second phase was significantly reduced by pretreatment with NK1, NK2, or CGRP receptor antagonists, suggesting that the secondary phase is neurogenically mediated.^2^

The neuroendocrine system through the release of CGRP and SP may play a role in the pathogenesis of sepsis.^104^ High systemic CGRP levels were associated with lethal outcome already at the onset of sepsis, whereas high SP levels were identified as late predictive indicators of lethal outcome.^104^

Onuoha and Alpar^38^ examined the concentration of sensory peptides in human thermal injuries, on admission and 24 hours postadmission, and their role in the metabolic, immunological, and inflammatory complications. Basal levels of CGRP and SP were significantly higher in patients with burn injuries than in the healthy control subjects.^38^ These results support the concept that the neuroendocrine system through the release of CGRP may play a critical role in the pathogenesis of sepsis.^12^,^38^,^104^

## CONCLUSIONS AND FUTURE PERSPECTIVES

This review has summarized, and attempted to correlate, the inflammatory activities of CGRP in burns. There have been previous reviews on the cardiovascular activities of CGRP^71^,^80^,^81^ that relate to this fascinating peptide family.^76^,^78^ Its most important activity is its potency in peripheral vasodilation.^44^,^125^–^127^ Several studies have shown that sensory peptides are released from peripheral nerve endings during a noxious or thermal stimulus, such as a scald, and may thus contribute to the pathophysiology of burn injuries.^12^,^38^,^103^,^119^–^124^ This suggests that, as described in this review, it probably plays an important role in the regulation of tissue perfusion, inflammation, and healing and tissue repair.^12^,^38^,^103^,^119^–^124^ More information regarding the concentration of CGRP in plasma in human burns is needed. It will be interesting to follow plasma concentration of CGRP in patients with burn injuries, from injury to healing, analyzing the burn for extension, location, and complication (sepsis, organ failure).

This may explain the impact of neurogenic inflammation in burn shock, and perhaps CGRP levels can be used as a prognostic factor in the clinical setting. In this way, it may be possible in the future to modulate the systemic response to burn to improve burn care. In recent years, the synthesis of nonpeptides that are capable of antagonizing effects mediated via the CGRP receptors has been a major advance.

## Figures and Tables

**Figure 1:**
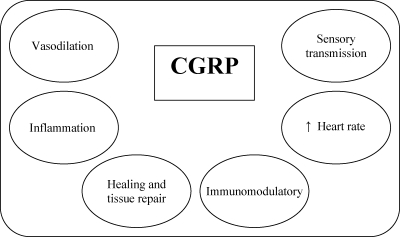
Relevance of calcitonin gene related peptide. Action in tissue repair.

**Table 1:** Inflammatory mediators of burn

Mediators	Effects	Tissue effect	References
Histamine	↓Blood pressure Hypovolemia	Arteriolar dilation and venular constriction ↑Blood flow ↑Permeability	15–18
Prostaglandin E_2_	↓Systemic arterial and pulmonary arterial blood pressure	Vasodilation ↑Blood flow ↑Permeability	19, 20
Prostacyclin (PGI_2_)	↓Blood pressure	↑Permeability	21
Leukotriene B_4_ Leukotriene D_4_	Pulmonary hypertension	Vasoconstriction of pulmonary vessels	21
Tromboxane A_2_ Tromboxane B_2_	GI* ischemia Pulmonary hypertension	Vasoconstriction ↑Permeability	19, 22–24
Bradykinin	↓Blood pressure Hypovolemia	Vasodilation ↑Permeability	21, 25
Serotonin		↑Permeability	18
Catecholamine	↑Heart rate ↑Blood pressure ↑Metabolism	Vasoconstriction (α receptors) Vasodilation (β_2_ receptors in muscle) Block ↑ permeability due to histamine and bradykinin (β receptors)	17, 21, 26
Oxygen radicals	Cardiac dysfunction	Tissue damage ↑Permeability	15, 16, 21, 27
Platelet aggregation factor	↑Blood pressure	Vasoconstriction	28–30
Angiotensin II	GI ischemia ↑Blood pressure	Vasoconstriction	31
Vasopressin	GI ischemia ↑Blood pressure	Vasoconstriction	32
Procalcitonin			33, 34
Antimicrobial peptides (defensins and cathelicidins)	Protective role against microbes		35–37
Tachykinins Substance P Neurokinin A Neurokinin B	Edema	Vasodilation ↑Permeability	38–43
Calcitonin gene–related peptide	↑Heart rate ↓Blood pressure	Vasodilation ↑Permeability Proliferative effect on human endothelium	38, 44

*GI indicates gastrointestinal.

## References

[1] Lund T, Onarheim H, Reed RK (1992). Pathogenesis of edema formation in burn injuries. World J Surg.

[2] Löfgren O, Qi Y, Lundeberg T, Gazelius B (1998). Antagonists of sensory neuropeptides inhibit the secondary phase of increased circulation following thermally induced inflammation. Microvasc Res.

[3] Amara SG, Jonas V, Rosenfeld MG (1982). Alternative RNA processing in calcitonin gene expression generates mRNA encoding different polypeptide products. Nature.

[4] Rosenfeld MG, Mermod JJ, Amara SG (1983). Production of a novel neuropeptide encoded by the calcitonin gene via tissue-specific RNA processing. Nature.

[5] Brain SD, Williams TJ, Tippins JR, Morris HR, MacIntyre I (1985). Calcitonin gene-related peptide is a potent vasodilator. Nature.

[6] Gherardini G, Jernbeck J, Samuelson U, Heden P (1995). Effects of calcitonin gene-related peptide and lidocaine on mechanically induced vasospasm in an island flap in the rat. J Reconstr Microsurg.

[7] Franco-Cereceda A, Gennari C, Nami R (1987). Cardiovascular effects of calcitonin gene-related peptides I and II in man. Circ Res.

[8] Samuelson UE, Jernbeck J (1991). Calcitonin gene-related peptide relaxes porcine arteries via one endothelium-dependent and one endothelium-independent mechanism. Acta Physiol Scand.

[9] McEwan J, Larkin S, Davies G (1986). Calcitonin gene-related peptide: a potent dilator of human epicardial coronary arteries. Circulation.

[10] Gennari C, Fischer JA (1985). Cardiovascular action of calcitonin gene-related peptide in humans. Calcif Tissue Int.

[11] Gherardini G, Lundeberg T, Matarasso A (1995). Calcitonin gene-related peptide increases microcirculation after mechanically induced ischemia in an experimental island flap. Ann Plast Surg.

[12] Gomes RN, Castro-Faria-Neto HC, Bozza PT (2005). Calcitonin gene-related peptide inhibits local acute inflammation and protects mice against lethal endotoxemia. Shock.

[13] Demling RH, Mazess RB, Witt RM, Wolberg WH (1978). The study of burn wound edema using dichromatic absorptiometry. J Trauma.

[14] Jakobsson OP, Benediktsson G, Arturson G (1985). Early post-burn oedema in leucocyte-free rats. Burns Incl Therm Inj.

[15] Till GO, Guilds LS, Mahrougui M, Friedl HP, Trentz O, Ward PA (1989). Role of xanthine oxidase in thermal injury of skin. Am J Pathol.

[16] Friedl HP, Till GO, Trentz O, Ward PA (1989). Roles of histamine, complement and xanthine oxidase in thermal injury of skin. Am J Pathol.

[17] Dyess DL, Hunter JL, Lakey JR, Moyer D, Dougherty FC, Townsley MI (2000). Attenuation of histamine-induced endothelial permeability responses after pacing-induced heart failure: role for endogenous catecholamines. Microcirculation.

[18] Carvajal HF, Brouhard BH, Linares HA (1975). Effect of antihistamine-antiserotonin and ganglionic blocking agents upon increased capillary permeability following burn trauma. J Trauma.

[19] Heggers JP, Loy GL, Robson MC, Del Beccaro EJ (1980). Histological demonstration of prostaglandins and thromboxanes in burned tissue. J Surg Res.

[20] Anggård E, Jonsson CE (1971). Efflux of prostaglandins in lymph from scalded tissue. Acta Physiol Scand.

[21] Goodman-Gilman A, Rall TW, Nies AS (1990). The Pharmacological Basis of Therapeutics.

[22] Huang YS, Li A, Yang ZC (1990). Roles of thromboxane and its inhibitor anisodamine in burn shock. Burns.

[23] LaLonde C, Demling RH (1989). Inhibition of thromboxane synthetase accentuates hemodynamic instability and burn edema in the anesthetized sheep model. Surgery.

[24] Tokyay R, Loick HM, Traber DL, Heggers JP, Herndon DN (1992). Effects of thromboxane synthetase inhibition on postburn mesenteric vascular resistance and the rate of bacterial translocation in a chronic porcine model. Surg Gynecol Obstet.

[25] Paul W, Douglas GJ, Lawrence L (1994). Cutaneous permeability responses to bradykinin and histamine in the guinea-pig: possible differences in their mechanism of action. Br J Pharmacol.

[26] Ding Z, Jiang M, Li S, Zhang Y (1995). Vascular barrier-enhancing effect of an endogenous beta-adrenergic agonist. Inflammation.

[27] Horton JW, White DJ (1995). Role of xanthine oxidase and leukocytes in postburn cardiac dysfunction. J Am Coll Surg.

[28] Wallace JL, Steel G, Whittle BJ, Lagente V, Vargaftig B (1987). Evidence for platelet-activating factor as a mediator of endotoxin-induced gastrointestinal damage in the rat. Effects of three platelet-activating factor antagonists. Gastroenterology.

[29] Ono I, Gunji H, Hasegawa T, Harada H, Kaneko F, Matsuzaki M (1993). Effects of a platelet activating factor antagonist on oedema formation following burns. Burns.

[30] Lu Z, Wolf MB (1993). Platelet activating factor-induced microvascular permeability increases in the cat hindlimb. Circ Shock.

[31] Sun K, Gong A, Wang CH, Lin BC, Zhu HN (1990). Effect of peripheral injection of arginine vasopressin and its receptor antagonist on burn shock in the rat. Neuropeptides.

[32] Cartotto R, McGibney K, Smith T, Abadir A (2007). Vasopressin for the septic burn patient. Burns.

[33] Lavrentieva A, Kontakiotis T, Lazaridis L (2007). Inflammatory markers in patients with severe burn injury. What is the best indicator of sepsis?. Burns.

[34] Bargues L, Chancerelle Y, Catineau J, Jault P, Carsin H (2007). Evaluation of serum procalcitonin concentration in the ICU following severe burn. Burns.

[35] Milner SM, Bhat S, Buja M, Gulati S, Poindexter BJ, Bick RJ (2004). Expression of human beta defensin 2 in thermal injury. Burns.

[36] Milner SM, Cole A, Ortega MR (2003). Inducibility of HBD-2 in acute burns and chronic conditions of the lung. Burns.

[37] Milner SM, Ortega MR (1999). Reduced antimicrobial peptide expression in human burn wounds. Burns.

[38] Onuoha GN, Alpar EK (2001). Levels of vasodilators (SP, CGRP) and vasoconstrictor (NPY) peptides in early human burns. Eur J Clin Invest.

[39] Sio SW, Puthia MK, Lu J, Moochhala S, Bhatia M (2008). J The neuropeptide substance P is a critical mediator of burn-induced acute lung injury. Immunology.

[40] Pernow B (1983). Substance P. Pharmacol Rev.

[41] Muangman P, Tamura RN, Muffley LA (2009). Substance P enhances wound closure in nitric oxide synthase knockout mice. J Surg Res.

[42] Lembeck F, Donnerer J, Tsuchiya M, Nagahisa A (1992). The non-peptide tachykinin antagonist, CP-96,345, is a potent inhibitor of neurogenic inflammation. Br J Pharmacol.

[43] Hughes SR, Williams TJ, Brain SD (1990). Evidence that endogenous nitric oxide modulates oedema formation induced by substance P. Eur J Pharmacol.

[44] Gherardini G, Gürlek A, Milner SM (1998). Calcitonin gene-related peptide improves skin flap survival and tissue inflammation. Neuropeptides.

[45] Bayliss WM (1901). On the origin from the spinal cord of the vasodilator fibres of the hind-limb, and on the nature of these fibres. J Physiol.

[46] Hoheisel U, Mense S, Scherotzke R (1994). Calcitonin gene-related peptide-immunoreactivity in functionally identified primary afferent neurones in the rat. Anat Embryol (Berl).

[47] Jänig W, Lisney SJ (1989). Small diameter myelinated afferents produce vasodilatation but not plasma extravasation in rat skin. Physiology.

[48] Holzer P, Lippe IT (1988). Stimulation of afferent nerve endings by intragastric capsaicin protects against ethanol-induced damage of gastric mucosa. Neuroscience.

[49] Brain SD, Hughes SR, Cambridge H, O'Driscoll G (1993). The contribution of calcitonin gene-related peptide (CGRP) to neurogenic vasodilator responses. Agents Actions.

[50] Gamse R, Posch M, Saria A, Jancsó G (1987). Several mediators appear to interact in neurogenic inflammation. Acta Physiol Hung.

[51] Lembeck F, Donnerer J, Barthó L (1982). Inhibition of neurogenic vasodilation and plasma extravasation by substance P antagonists, somatostatin and [D-Met2, Pro5]enkephalinamide. Eur J Pharmacol.

[52] Lundberg JM, Alving K, Karlsson JA, Matran R, Nilsson G (1991). Sensory neuropeptide involvement in animal models of airway irritation and of allergen-evoked asthma. Am Rev Respir Dis.

[53] Maggi CA (1991). The pharmacology of the efferent function of sensory nerves. J Auton Pharmacol.

[54] Said SI (1990). Neuropeptides as modulators of injury and inflammation. Life Sci.

[55] Lippe IT, Stabentheiner A, Holzer P (1993). Role of nitric oxide in the vasodilator but not exudative component of mustard oil-induced inflammation in rat skin. Agents Actions.

[56] Hughes SR, Brain SD (1994). Nitric oxide-dependent release of vasodilator quantities of calcitonin gene-related peptide from capsaicin-sensitive nerves in rabbit skin. Br J Pharmacol.

[57] Kajekar R, Moore PK, Brain SD (1995). Essential role for nitric oxide in neurogenic inflammation in rat cutaneous microcirculation. Evidence for an endothelium-independent mechanism. Circ Res.

[58] Geppetti P (1993). Sensory neuropeptide release by bradykinin: mechanisms and pathophysiological implications. Regul Pept.

[59] Holzer P (1991). Capsaicin as a tool for studying sensory neuron functions. Adv Exp Med Biol.

[60] Couture R, Laneuville O, Guimond C, Drapeau G, Regoli D (1989). Characterization of the peripheral action of neurokinins and neurokinin receptor selective agonists on the rat cardiovascular system. Naunyn Schmiedebergs Arch Pharmacol.

[61] Ralevic V, Mathie RT, Alexander B, Burnstock G (1991). NG-nitro-L-arginine methyl ester attenuates vasodilator responses to acetylcholine but enhances those to sodium nitroprusside. J Pharm Pharmacol.

[62] Couture R, Cuello AC (1984). Studies on the trigeminal antidromic vasodilatation and plasma extravasation in the rat. J Physiol.

[63] Maggi CA (1995). Tachykinins and calcitonin gene-related peptide (CGRP) as co-transmitters released from peripheral endings of sensory nerves. Prog Neurobiol.

[64] Church MK, Lowman MA, Robinson C, Holgate ST, Benyon RC (1989). Interaction of neuropeptides with human mast cells. Int Arch Allergy Appl Immunol.

[65] Pearce FL, Kassessinoff TA, Liu WL (1989). Characteristics of histamine secretion induced by neuropeptides: implications for the relevance of peptide-mast cell interactions in allergy and inflammation. Int Arch Allergy Appl Immunol.

[66] Coderre TJ, Basbaum AI, Levine JD (1989). Neural control of vascular permeability: interactions between primary afferents, mast cells, and sympathetic efferents. J Neurophysiol.

[67] Piotrowski W, Devoy MA, Jordan CC, Foreman JC (1984). The substance P receptor on rat mast cells and in human skin. Agents Actions.

[68] Morris HR, Panico M, Etienne T, Tippins J, Girgis SI, MacIntyre I (1984). Isolation and characterization of human calcitonin gene-related peptide. Nature.

[69] Inzerillo AM, Zaidi M, Huang CL (2002). Calcitonin: the other thyroid hormone. Thyroid.

[70] Hoff AO, Catala-Lehnen P, Thomas PM (2002). Increased bone mass is an unexpected phenotype associated with deletion of the calcitonin gene. J Clin Invest.

[71] Brain SD, Grant AD (2004). Vascular actions of calcitonin gene-related peptide and adrenomedullin. Physiol Rev.

[72] Feuerstein G, Willette R, Aiyar N (1995). Clinical perspectives of calcitonin gene related peptide pharmacology. Can J Physiol Pharmacol.

[73] Holzer P (1995). Chemosensitive afferent nerves in the regulation of gastric blood flow and protection. Adv Exp Med Biol.

[74] Muff R, Born W, Fischer JA (1995). Calcitonin, calcitonin gene-related peptide, adrenomedullin and amylin: homologous peptides, separate receptors and overlapping biological actions. Eur J Endocrinol.

[75] Poyner D (1995). Pharmacology of receptors for calcitonin gene-related peptide and amylin. Trends Pharmacol Sci.

[76] Van Rossum D, Hanisch UK, Quirion R (1997). Neuroanatomical localization, pharmacological characterization and functions of CGRP, related peptides and their receptors. Neurosci Biobehav Rev.

[77] Wimalawansa SJ (1996). Calcitonin gene-related peptide and its receptors: molecular genetics, physiology, pathophysiology, and therapeutic potentials. Endocr Rev.

[78] Wimalawansa SJ (1997). Amylin, calcitonin gene-related peptide, calcitonin, and adrenomedullin: a peptide superfamily. Crit Rev Neurobiol.

[79] Brain SD, Williams TJ (1988). Substance P regulates the vasodilator activity of calcitonin gene-related peptide. Nature.

[80] Bell D, McDermott BJ (1996). Calcitonin gene-related peptide in the cardiovascular system: characterization of receptor populations and their (patho)physiological significance. Pharmacol Rev.

[81] Brain SD, Cambridge H (1996). Calcitonin gene-related peptide: vasoactive effects and potential therapeutic role. Gen Pharmacol.

[82] Marshall I (1992). Mechanism of vascular relaxation by the calcitonin gene-related peptide. Ann N Y Acad Sci.

[83] Crossman D, McEwan J, MacDermot J, MacIntyre I, Dollery CT (1987). Human calcitonin gene-related peptide activates adenylate cyclase and releases prostacyclin from human umbilical vein endothelial cells. Br J Pharmacol.

[84] Bouvier M (2001). Oligomerization of G-protein-coupled transmitter receptors. Nat Rev Neurosci.

[85] Crossman DC, Dashwood MR, Brain SD, McEwan J, Pearson JD (1990). Action of calcitonin gene-related peptide upon bovine vascular endothelial and smooth muscle cells grown in isolation and co-culture. Br J Pharmacol.

[86] Hirata Y, Takagi Y, Takata S, Fukuda Y, Yoshimi H, Fujita T (1998). Calcitonin gene-related peptide receptor in cultured vascular smooth muscle and endothelial cells. Biochem Biophys Res Commun.

[87] Reslerova M, Loutzenhiser R (1998). Renal microvascular actions of calcitonin gene-related peptide. Am J Physiol Renal Physiol.

[88] Sakai K, Saito K (1998). Reciprocal interactions among neuropeptides and adenosine in the cardiovascular system of rats: a role of K(ATP) channels. Eur J Pharmacol.

[89] Maggi CA, Santicioli P, Giuliani S (1995). Role of cyclic AMP and protein kinase A in K^+^ channel activation by calcitonin gene-related peptide (CGRP) in the guinea-pig ureter. J Auton Pharmacol.

[90] Pomerleau F, Fournier A, Cadieux A (1997). Mouse aorta: a preparation highly sensitive to the vasodilatory action of CGRP. J Cardiovasc Pharmacol.

[91] Raddino R, Pela G, Manca C (1997). Mechanism of action of human calcitonin gene-related peptide in rabbit heart and in human mammary arteries. J Cardiovasc Pharmacol.

[92] Sugo S, Minamino N, Kangawa K (1994). Endothelial cells actively synthesize and secrete adrenomedullin. Biochem Biophys Res Commun.

[93] Gray DW, Marshall I (1992). Human alpha-calcitonin gene-related peptide stimulates adenylate cyclase and guanylate cyclase and relaxes rat thoracic aorta by releasing nitric oxide. Br J Pharmacol.

[94] Gray DW, Marshall I (1992). Nitric oxide synthesis inhibitors attenuate calcitonin gene-related peptide endothelium-dependent vasorelaxation in rat aorta. Eur J Pharmacol.

[95] Seike M, Ikeda M, Morimoto A, Matsumoto M, Kodama H (2002). Increased synthesis of calcitonin gene-related peptide stimulates keratinocyte proliferation in murine UVB-irradiated skin. J Dermatol Sci.

[96] Nahin RL, Byers MR (1994). Adjuvant-induced inflammation of rat paw is associated with altered calcitonin gene-related peptide immunoreactivity within cell bodies and peripheral endings of primary afferent neurons. J Comp Neurol.

[97] Buckley TL, Brain SD, Rampart M, Williams TJ (1991). Time-dependent synergistic interactions between the vasodilator neuropeptide, calcitonin gene-related peptide (CGRP) and mediators of inflammation. Br J Pharmacol.

[98] Buckley TL, Brain SD, Jose PJ, Williams TJ (1992). The partial inhibition of inflammatory responses induced by capsaicin using the Fab fragment of a selective calcitonin gene-related peptide antiserum in rabbit skin. Neuroscience.

[99] Donnerer J, Schuligoi R, Stein C (1992). Increased content and transport of substance P and calcitonin gene-related peptide in sensory nerves innervating inflamed tissue: evidence for a regulatory function of nerve growth factor in vivo. Neuroscience.

[100] Alving K, Sundström C, Matran R, Panula P, Hökfelt T, Lundberg JM (1991). Association between histamine-containing mast cells and sensory nerves in the skin and airways of control and capsaicin-treated pigs. Cell Tissue Res.

[101] Lowman MA, Benyon RC, Church MK (1988). Characterization of neuropeptide-induced histamine release from human dispersed skin mast cells. Br J Pharmacol.

[102] Dalsgaard CJ, Jonsson CE, Haegerstrand A, Brodin E (1987). Sensory neuropeptides contribute to oedema formation in experimental burns. Scand J Plast Reconstr Surg Hand Surg.

[103] Onuoha GN, Alpar EK (1999). Calcitonin gene-related peptide and other neuropeptides in the plasma of patients with soft tissue injury. Life Sci.

[104] Beer S, Weighardt H, Emmanuilidis K (2002). Systemic neuropeptide levels as predictive indicators for lethal outcome in patients with postoperative sepsis. Crit Care Med.

[105] Kjatansson J, Dalsgaard GJ, Janssen CE (1987). Decreased survival of experimental critical skin flaps in rats after sensory denervation with capsaicin. Plast Reconstr Surg.

[106] Haegerstrand A, Dalsgaard CJ, Jonzon B, Larsson O, Nilsson J (1990). Calcitonin gene-related peptide stimulates proliferation of human endothelial cells. Proc Natl Acad Sci U S A.

[107] Karanth SS, Dhital S, Springall DR, Polak JM (1990). Reinnervation and neuropeptides in mouse skin flaps. J Auton Nerv Syst.

[108] Greenberg B, Rhoden K, Barnes P (1987). Calcitonin gene-related peptide (CGRP) is a potent non-endothelium-dependent inhibitor of coronary vasomotor tone. Br J Pharmacol.

[109] Torii H, Hosoi J, Beissert S (1997). Regulation of cytokine expression in macrophages and the Langerhans cell-like line XS52 by calcitonin gene-related peptide. J Leukoc Biol.

[110] Liu J, Chen M, Wang X (2000). Calcitonin gene-related peptide inhibits lipopolysaccharide-induced interleukin-12 release from mouse peritoneal macrophages, mediated by the cAMP pathway. Immunology.

[111] Sevitt S (1954). Pathological sequelae of burns, local vascular changes in burned skin. Proc R Soc Med.

[112] Arturson G (1961). Pathophysiological aspects of the burn syndrome with special reference to liver injury and alterations of capillary permeability. Acta Chir Scand Suppl.

[113] Jancsó N, Jancsó-Gábor A, Szolcsányi J (1967). Direct evidence for neurogenic inflammation and its prevention by denervation and by pretreatment with capsaicin. Br J Pharmacol Chemother.

[114] Saria A (1984). Substance P in sensory nerve fibres contributes to the development of oedema in the rat hind paw after thermal injury. Br J Pharmacol.

[115] Fleischer E, Handwerker HO, Joukhadar S (1983). Unmyelinated nociceptive units in two skin areas of the rat. Brain Res.

[116] Sevitt S (1958). Early and delayed oedema and increase in capillary permeability after burns of the skin. J Pathol Bacteriol.

[117] Saria A, Lundberg JM (1983). Capsaicin pretreatment inhibits heat-induced oedema in the rat skin. Naunyn Schmiedebergs Arch Pharmacol.

[118] Lundberg JM, Saria A, Rosell S, Folkers KA (1984). Substance P antagonist inhibits heat-induced oedema in the rat skin. Acta Physiol Scand.

[119] Haegerstrand A, Dalsgaard CJ, Jonsson CE (1987). Effects of capsaicin pretreatment on the inflammatory response to scalding injury in the rat. Acta Physiol Scand.

[120] Kandel ER, Schwartz JH, Jessell TM (2000). The Perception of Pain in Principles of Neural Science.

[121] Blomgren I, Bagge U (1984). Postburn blood flow, edema, and survival of the hairy mouse ear after scald injury at different temperatures. Scand J Plast Reconstr Surg.

[122] Siney L, Brain SD (1996). Involvement of sensory neuropeptides in the development of plasma extravasation in rat dorsal skin following thermal injury. Br J Pharmacol.

[123] Löfgren O, Yu LC, Theodorsson E, Hansson P, Lundeberg T (1997). Intrathecal CGRP(8-37) results in a bilateral increase in hindpaw withdrawal latency in rats with a unilateral thermal injury. Neuropeptides.

[124] Löfgren O, Palmer B, Theodorsson E, Törkvist L, Lundeberg T (1998). Contribution of the sensory and sympathetic nervous system to scalding-induced edema in the rat paw. Burns.

[125] Gherardini G, Evans GR, Theodorsson E (1996). Calcitonin gene-related peptide in experimental ischemia. Implication of an endogenous anti-ischemic effect. Ann Plast Surg.

[126] Gherardini G, Evans GR, Milner SM (1996). Comparison of vascular effects of calcitonin gene-related peptide and lidocaine on human veins. J Reconstr Microsurg.

[127] Gherardini G, Gürlek A, Evans GR (1998). Venous ulcers: improved healing by iontophoretic administration of calcitonin gene-related peptide and vasoactive intestinal polypeptide. Plast Reconstr Surg.

